# 3,4-Dihydroxyphenylethanol (DPE or Hydroxytyrosol) Counteracts ERK1/2 and mTOR Activation, Pro-Inflammatory Cytokine Release, Autophagy and Mitophagy Reduction Mediated by Benzo[a]pyrene in Primary Human Colonic Epithelial Cells

**DOI:** 10.3390/pharmaceutics14030663

**Published:** 2022-03-17

**Authors:** Roberta Santarelli, Chiara Pompili, Maria Saveria Gilardini Montani, Lorenzo Evangelista, Roberta Gonnella, Mara Cirone

**Affiliations:** 1 Department of Experimental Medicine, “Sapienza” University of Rome, 00185 Rome, Italy; roberta.santarelli@uniroma1.it (R.S.); chiarapompili08@gmail.com (C.P.); mariasaveria.gilardinimontani@uniroma1.it (M.S.G.M.); evangelista.1806844@studenti.uniroma1.it (L.E.); roberta.gonnella@uniroma1.it (R.G.); 2 Laboratory affiliated to Istituto Pasteur Italia-Fondazione Cenci Bolognetti, 00185 Rome, Italy

**Keywords:** Benzo[a]pyrene, 3,4-Dihydroxyphenylethanol, Hydroxytyrosol, human colonic epithelial cells, autophagy, ERK, mTOR, p21, inflammation, colon cancer

## Abstract

Understanding the effects induced by carcinogens on primary colonic epithelial cells and how to counteract them might help to prevent colon cancer, which is one of the most frequent and aggressive cancers. In this study, we exposed primary human colonic epithelial cells (HCoEpC) to Benzo[a]pyrene (B[a]P) and found that it led to an increased production of pro-inflammatory cytokines and activated ERK1/2 and mTOR. These pathways are known to be involved in inflammatory bowel disease (IBD), which represents a colon cancer risk factor. Moreover, B[a]P reduced autophagy and mitophagy, processes whose dysregulation has been clearly demonstrated to predispose to cancer either by in vitro or in vivo studies. Interestingly, all the effects induced by B[a]P could be counteracted by 3,4-Dihydroxyphenylethanol (DPE or Hydroxytyrosol, H), the most powerful anti-inflammatory and antioxidant compound contained in olive oil. This study sheds light on the mechanisms that could be involved in colon carcinogenesis induced by a chemical carcinogen and identifies a safe natural product that may help to prevent them.

## 1. Introduction

It has been now clearly ascertained that chronic inflammation represents a risk factor for the development of cancer, as about 20% of all human malignancies seem to be linked to chronic inflammatory conditions [1]. Regarding the colon, it is known that inflammatory bowel diseases (IBDs), which include Chron’s disease and ulcerative colitis, are syndromes predisposing to colon cancer onset [2]. Moreover, hereditary non-polyposis colorectal cancer (HNPCC, also called Lynch syndrome) and familial adenomatous polyposis (FAP) syndrome [3] are disorders predisposing to a high risk of colon cancer development.

The exposure to environmental and food-borne mutagens plays an important role in colon cancer development [4]. Benzo[a]pyrene (B[a]P), a polycyclic aromatic hydrocarbon (PAH) found in the environment as well as in food, particularly in fried and grilled aliments, is classified as a Group I human carcinogen by the International Agency for Research on Cancer (IARC) [5]. B[a]P, besides being a lung carcinogen [6], can be considered a carcinogen for the colon [7]. As one of the underlying mechanisms leading to B[a]P-driven tumorigenesis, it is known that its metabolites can directly induce the formation of DNA adducts [8]. Of note, carcinogens may cooperate with inflammation to promote carcinogenesis [9] and may themselves trigger an inflammatory response [10]. Accordingly, it has been reported that B[a]P is able to increase the release of interleukin (IL)-1beta, IL-6 and tumor necrosis factor (TNF) alpha in animal models and that such effects could be counteracted by the flavonoid Quercetin [11]. Interestingly, another in vivo study has demonstrated that B[a]P-driven colon carcinogenesis could be prevented by olive oil, the king nutrient of the Mediterranean diet [12]. Olive oil contains several potent antioxidant and anti-inflammatory compounds, among which the most powerful one is represented by 3,4-Dihydroxyphenylethanol (DPE or Hydroxytyrosol, H) [12]. However, the in vivo studies evidencing the carcinogenic potential of B[a]P and the anti-tumorigenic effects of natural compounds do not completely elucidate the molecular mechanisms through which they mediate such effects. Hence, in this study, we exposed for the first time primary human colonic epithelial cells (HCoEpC) to B[a]P and evaluated its impact on cell survival and on the release of pro-inflammatory cytokines and chemokines, molecules able to recruit monocytes and B lymphocytes, which could contribute to inflammation, angiogenesis and tumorigenesis. We also assessed whether the release of cytokines or chemokines correlated with the activation of pro-inflammatory pathways such as extracellular signal-regulated protein kinase 1/2 (ERK1/2) and mammalian target of rapamycin (mTOR). Indeed, both have been shown to play an important role in IBD [13,14,15], which, as previously mentioned, strongly increases colon cancer risk [16]. Besides, we wondered if Hydroxytyrosol could counteract all the observed B[a]P-mediated effects. Finally, autophagy and mitophagy were investigated in B[a]P-treated colonic epithelial cells, in the presence or in the absence of Hydroxytyrosol, since these degradative processes influence and are influenced by inflammatory cytokines [17,18], can be regulated by mTOR [19] and ERK1/2 activation [20,21] and, if dysregulated, may favor colorectal carcinogenesis [22].

## 2. Materials and Methods

### 2.1. Cell Cultures, Reagents and Treatments

Human colonic epithelial cells (HCoEpC) were purchased from iXCells Biotechnologies. HCoEpC were cultured in Epithelial Cell Growth Medium (iXCells Biotechnologies, Cat# MD-0041) at 37 °C in a 5% CO_2_ incubator. In 2D experiments, HCoEpC were seeded at a density of 5 × 10^4^ cells/well in 6-well plate and treated with 5 µM Benzo[a]pyrene (B[a]P) (Sigma-Aldrich, Cat# 48564, St. Louis, MO, USA). Depending on the experiment:

− Cells were grown for 96 h and 6 days, adding Benzo[a]pyrene every other day;
− Pre-treatments with 1 µM Hydroxytyrosol (H) (MedChemExpress, Cat# HY-N0570, Monmouth Junction, NJ 08852, USA) were performed for 45 min before adding B[a]P;
− HCoEpC were incubated with 1 µM UC2288 (Calbiochem- Sigma-Aldrich, Cat# 532813, St. Louis, MO, USA), a p21 inhibitor, for 6 days, refreshing medium supplemented with the inhibitor every other day;
− In order to evaluate autophagy, cells treated or not (CT) with Benzo[a]pyrene for 6 days were incubated or not with 20 nM Bafilomycin A1 (MedChemExpress, Cat# HY-100558, Monmouth Junction, NJ 08852, USA) for the last 4 h.

HCoEpC 3D cultures were established by seeding 3 × 10^3^ cells/well in BIOFLOAT^TM^ 96-well plates provided by faCellitate. After seeding, plates were centrifuged at 500× *g* for 5 min and, subsequently, maintained at 37 °C in a 5% CO_2_ incubator to allow spheroid growth. Then, spheroids were tested under the same experimental conditions of 2D cultures.

DMSO was added as vehicle in untreated control cells, in all the experiments.

### 2.2. Cell Viability Assay

To assess cell viability, 1 × 10^3^ cells/well were seeded in 96-well plates and treated with 5, 10 and 20 µM B[a]P for 96 h and 6 days; in the presence or absence of 1 µM UC2288 for 6 days; 1 µM Hydroxytyrosol in the presence or absence of 5µM B[a]P. Cell viability was assayed using a cell proliferation kit, MTT, (Roche), following the manufacturer’s instructions. Absorbance was evaluated by Absorbance 96 reader (Byonoy GmbH, Hamburg, Germany). 

### 2.3. Chemiluminescent Immunometric Assay

Supernatants from HCoEpC, untreated (CT) and treated with Benzo[a]pyrene and/or Hydroxytyrosol for 6 days, were collected and Interleukin-6 (IL-6), Interleukin-8 (IL-8), vascular endothelial growth factor (VEGF), CXCL13, CCL2 and Cathepsin S were measured by Magnetic Luminex assay using a human premixed multi-analyte kit (R&D Systems Bio-Techne, LXSAHM, Minneapolis, MN, USA), following the manufacturer’s instructions. 

### 2.4. Monocyte Isolation and Macrophage Differentiation

Monocytes isolated from human peripheral blood mononuclear cells (PBMCs) of healthy donors, as previously described [23], were cultured in RPMI 1640 (Euroclone, Cat# ECB9006L, Milano, Italy) containing 10% FCS, 2 mM L-glutamine, 100 U/mL penicillin and 100 mg/mL streptomycin (complete medium) with the addition of 50 ng/mL recombinant human macrophage-colony stimulating factor M-CSF (Peprotech, Cat# 300-25, Waltham, MA, USA) every two days for 6/7 days to differentiate in macrophages.

### 2.5. Immunofluorescence Staining and FACS Analysis

For immunofluorescence, 1 × 10^6^ macrophages were cultured in a 24-well plate with (25% vol) supernatants from HCoEpC treated with B[a]P or not (CT). Supernatants were concentrated by centrifuge using Microcon-10 kDa Centrifugal Filters (Millipore) according to the manufacturer’s instructions, subsequently resuspended in RPMI and then added to macrophages cultures. After 24 h, cells were washed and incubated for 30 min at 4 °C with CD86 antibody (Miltenyi Biotec, Cat# 130-094-878, Bergisch Gladbach, Germany). Cells were analyzed with FACSCalibur, using CELLQuest software (BD Biosciences, Franklin Lakes, NJ, USA). Cells were gated according to their forward scatter and side scatter properties. At least 10 × 10^3^ events were acquired for each sample.

### 2.6. Western Blotting

Cells in 2D cultures were detached with Accutase (Capricorn Scientific, Ebsdorfergrund, Germany) for 10 min at 37 °C in cell incubator, while spheroids in 3D cultures were harvested and then washed twice in PBS. Cell pellets were lysed in ice in RIPA buffer (NaCl 150 mM, NP40 1%, Tris-HCl pH8 50 mM, deoxycholic acid 0.5%, SDS 0.1%) containing protease and phosphatase inhibitors. A total of 10 µg of proteins for each sample was loaded on a precast polyacrylamide gel (Bolt^TM^ 4–12% Bis-Tris Plus, Invitrogen, Waltham, MA, USA) and transferred to a nitrocellulose membrane (Amersham ^TM^). Membranes were incubated for 30 min in a blocking solution (PBS, Tween-20 0.1%, BSA 2%) and then with primary antibodies for 1 h at room temperature or overnight at 4 °C. Membranes were washed three times in PBS Tween-20 0.1% and then incubated for 30 min with the proper secondary antibody conjugated to horseradish peroxidase. Then, membranes were washed three times in PBS Tween-20 0.1% and protein detection was performed through a chemiluminescence kit WesternBright ECL (Advansta, Menio Park, CA, USA). Densitometric analysis was carried out using ImageJ software (https://imagej.nih.gov/ij/ (accessed on 13 January 2022)).

### 2.7. Antibodies

All primary and secondary antibodies were diluted in PBS Tween-20 0.1% containing BSA 2%. Primary antibodies used were: rabbit polyclonal anti-HADHA (Proteintech, Cat# 10758-1-ap) 1:500; rabbit polyclonal anti-SQSTM1/p62 (Novus, Cat# NBP-48320, Centennial, CO, USA) 1:4000; mouse monoclonal anti-phosphoERK1/2 (Santa Cruz, Cat# 7383, Dallas, TX, USA) 1:500; rabbit polyclonal anti-ERK1 and anti-ERK2 (Santa Cruz, Cat# sc-93 and Cat# sc-154) 1:500; rabbit polyclonal anti-p21 (Proteintech, Cat# 10355-1-AP) 1:800; rabbit polyclonal anti-LC3 I/II (Novus, Cat# NB100-2220) 1:1000; rabbit monoclonal anti-phospho4EBP1 (Cell Signaling Technology, Cat# 2855T, Danvers, MA, USA) 1:500; mouse monoclonal anti-4EBP1 (Proteintech, Cat# 60246-1-1g) 1:500; mouse monoclonal anti-β Actina (Sigma Aldrich, Cat# A5441) 1:10,000.

Goat polyclonal anti-mouse 1:15,000 (Santa Cruz, Cat# sc-2005) and anti-rabbit 1:15,000 (Santa Cruz, Cat# sc-2004) were used as secondary antibodies.

#### 2.7.1. Indirect Immunofluorescence Assay (IFA)

To perform immunofluorescence, 2 × 10^4^ HCOEPC were seeded on cover glasses in 24-well plate and treated with Benzo[a]pyrene (5 µM) and/or Hydroxytyrosol (1 µM) for 6 days. Then, cells were washed in PBS and fixed in Paraformaldehyde 2% (Electron Microscopy Sciences, Hatfield, PA, USA) for 30 min. After washing in PBS, cells were permeabilised with Triton-X 100 0.15% for 5 min, washed in PBS and then incubated with BSA 3%-Glycine 1% (in PBS) for additional 30 min. Subsequently, cells were incubated with rabbit polyclonal anti-SQSTM1/p62 (Novus, Cat# NBP-48320; 1:200) primary antibody for 1 h at room temperature and then washed in PBS. Next, cells were incubated with Fluorescein (FITC)-conjugated AffiniPure goat anti-rabbit (Jackson Immuno Research Labs, Cat# 111-095-045; 1:100, West Grove, PA, USA) for 30 min and, following several washes in PBS, nuclei were stained with DAPI for 1 min. Subsequently, cover glasses were washed in PBS and mounted with PBS:Glycerol (1:1) on microscope slides. Finally, analysis was performed with Olympus BX53 (Shinjuku-ku, Tokyo, Japan) fluorescence microscope at 20× magnification.

#### 2.7.2. MitoTracker^TM^ Green-FM Staining

HCoEpC were grown on cover glasses, treated with Benzo[a]pyrene (5 µM) and/or Hydroxytyrosol (1 µM) for 6 days, and mitochondrial labelling was performed by adding MitoTracker^TM^ Green-FM (Invitrogen, Cat# M7514, MA, USA) fluorescent dye (200 nM) to cell culture medium for the last 30 min at 37 °C. Then, cells were washed in PBS and cover glasses mounted with PBS:Glycerol (1:1) on microscope slides. Cells were analyzed with Apotome Axio Observer Z1 inverted microscope (Zeiss, Munich, Germany) equipped with an AxioCam MRM Rev.3 (Germany) at 40× magnification.

### 2.8. Statistical Analysis

Results are shown as the mean ± standard deviation (SD) of three independent experiments. Statistical analysis was performed by Student’s *t*-test. Differences were considered significant if *p*-value was < 0.05 (*) and <0.01 (**).

## 3. Results

### 3.1. B[a]P Exerts a Cytotoxic Effect in Primary Colonic Epithelial Cells, Increases the Release of IL-6, VEGF, IL-8, CXCL13, Cathepsin S and Activates Macrophages 

The toxicity of B[a]P towards primary human colonic epithelial cells (HCoEpC) was first investigated. Indeed, although previous papers showed that this compound is cytotoxic to several cancer cell lines, primary cells [24,25] and in mouse model [26], no in vitro studies on primary human colonic epithelial cells have been performed yet.

**Figure 1:**
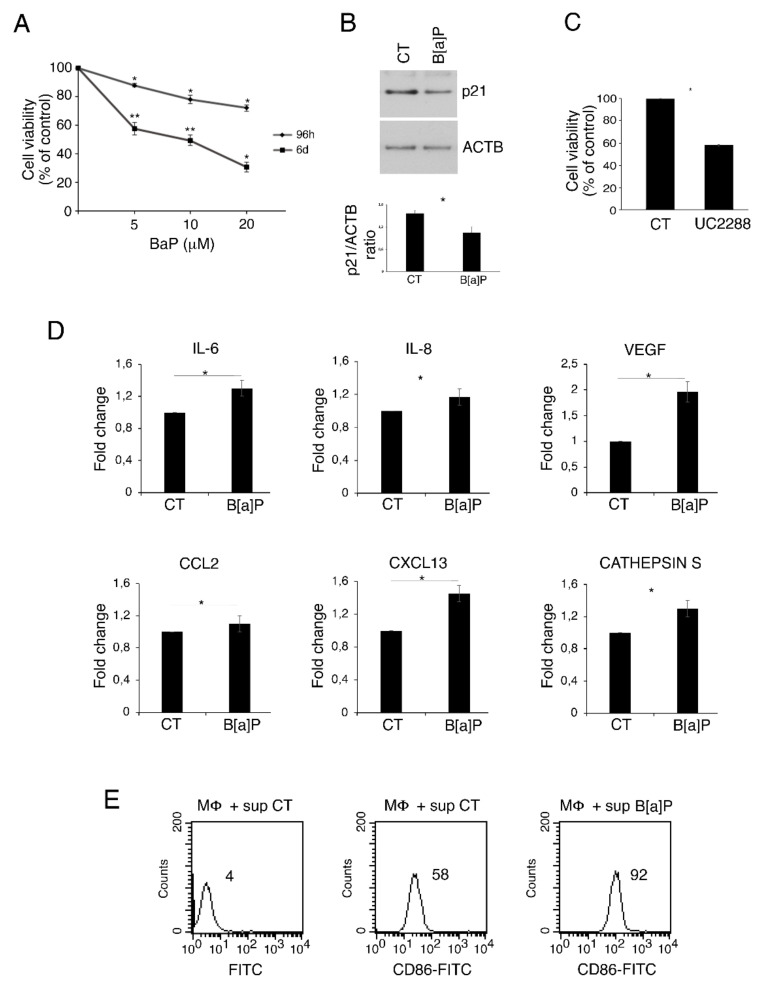
B[a]P reduces cell viability in HCoEpC through p21, induces pro-inflammatory cytokines and activates macrophages. (**A**) % of cell viability of HCoEpC treated with different B[a]P concentrations (5, 10 and 20 μM) for 96 h and 6 days evaluated by MTT assay. Mean ± standard deviation (SD) of three independent experiments is shown. (*) *p* < 0.05, (**) *p* < 0.01. (**B**) p21 level in HCoEpC treated with B[a]P (5 μM) for 6 days was assessed by Western blotting. Actin (ACTB) was used as loading control and one representative experiment out of three is shown. The histograms represent the mean plus SD of the densitometric analysis of the ratio p21/ACTB of three different experiments. (*) *p* < 0.05. (**C**) % of cell viability of HCoEpC treated with p21 inhibitor UC2288 (1 μM) for 6 days evaluated by MTT assay. Mean ± standard deviation (SD) of three independent experiments is shown. (*) *p* < 0.05. (**D**) Fold change relative to control of IL-6, IL-8, VEGF, CXCL13, CCL2 and Cathepsin S released by HCoEpC treated with B[a]P (5 μM) for 6 days. Mean ± standard deviation (SD) of three independent experiments is shown. (*) *p* < 0.05. (**E**) FACS analysis of CD86 expression on macrophages cultured in presence of CT and B[a]P-treated HCoEpC supernatants. A representative experiment is shown, and the mean of fluorescence intensity is indicated. FITC is the isotype control. Untreated cells were used as control (CT) in all the experiments in the figure.

For this purpose, we treated HCoEpC with different B[a]P concentrations (5–20 μM) for up to 6 days and evaluated cell viability by MTT assay. We observed a dose- and time-dependent reduction in cell survival compared to the untreated control (Figure 1A). Based on this result, we chose to perform next experiments with B[a]P 5 μM, since cell viability was about 58% at this concentration after 6 days of treatment. As shown by Western blotting, this cytotoxic effect mediated by B[a]P occurred in correlation with the reduction of p21 (Figure 1B), whose pro-survival role was demonstrated in this study by the use of p21 inhibitor UC2288 (1 μM) (Figure 1C). We then evaluated the impact of B[a]P on the release of pro-inflammatory cytokines and found an increase in IL-6 and vascular endothelial growth factor (VEGF) as well as of IL-8 and CXCL13 chemokines, while the release of CCL2 was slightly affected following the exposure of HCoEpC to 5 μM B[a]P for 6 days (Figure 1D). Interestingly, we also detected an increased Cathepsin S release (Figure 1D), which is an important finding as this lysosomal enzyme has been reported to contribute to angiogenesis and carcinogenesis [27], particularly in the case of colon cancer [28]. Given the strong link between inflammation and cancer, these results suggest that an increased production of pro-inflammatory cytokines, chemokines and Cathepsin S could contribute to B[a]P-driven carcinogenesis. Finally, we evaluated the impact of the cytokines released by B[a]P-treated HCoEpC on macrophage activation. As shown in Figure 1E, macrophages incubated in the presence of B[a]P-cell supernatant up-regulated CD86 activation marker, whose signaling has been reported to promote inflammatory cytokine production [29]. Hence, B[a]P could further promote colon inflammation through the recruitment and activation of macrophages.

### 3.2. B[a]P Activates ERK1/2 and mTOR in 2D and 3D Culture Models of HCoEpC 

**Figure 2:**
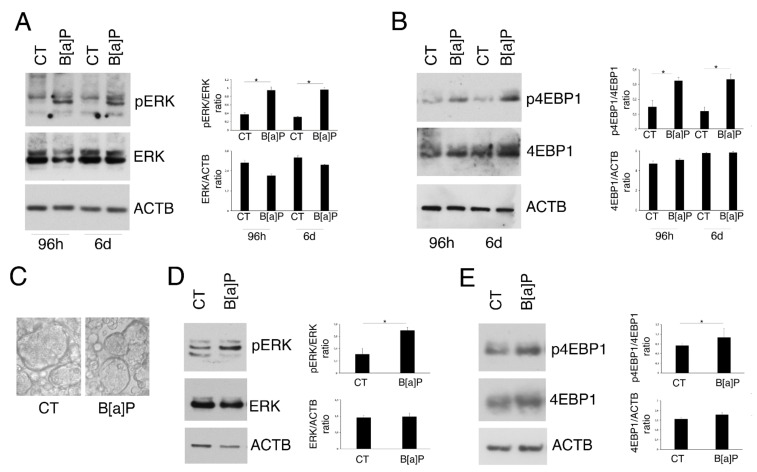
ERK and mTOR pathways are activated by B[a]P in HCoEpC cultured in 2D and 3D. (**A**) Phospho-ERK 1/2 (pERK) and (**B**) phospho-4EBP1 (p4EBP1) levels were evaluated in HCoEpC cultured in 2D and treated with B[a]P (5 μM) for 96 h and 6 days by Western blotting. (**C**) Representative image of 3D culture of HCoEpC (spheroids) untreated or treated with B[a]P (5 μM) for 6 days. Magnification 20×. (**D**) Western blotting analysis to assess phospho-ERK1/2 (pERK) and (**E**) phospho-4EBP1 (p4EBP1) levels in HCoEpC cultured in 3D and treated with B[a]P (5 μM) for 6 days. Actin (ACTB) was used as loading control and one representative experiment out of three is shown. The histograms represent the mean plus SD of the densitometric analysis of the ratio pERK/ERK, ERK/ACTB, p4EBP1/4EBP1 and 4EBP1/ACTB of three different experiments. (*) *p* < 0.05. Untreated cells were used as control (CT) in all the experiments in the figure.

Among the pro-inflammatory pathways activated in IBD and playing a key role in the control of cytokine release, there are ERK1/2 and PI3K/AKT/mTOR. Therefore, we evaluated the phosphorylation of ERK1/2 and 4EBP1 mTOR target, in HCoEpC exposed or not to 5 µM B[a]P for 96 h and 6 days. As shown in Figure 2A,B, ERK1/2, in particular ERK2, and 4EBP1 were activated by B[a]P at both time points. To use a model that could more closely mimic what may occur in vivo, we repeated the above-reported experiment on HCoEpC cultured in 3D (Figure 2C) and found that the phosphorylation of ERK1/2 and 4EBP1 also increased in these cells following B[a]P exposure, similar to the 2D cultures (Figure 2D,E). For this reason, all the next experiments have been performed in 2D.

### 3.3. Hydroxytyrosol Slightly Increases Cell Death and Counteracts Cytokine and Chemokine Release as Well as ERK1/2 and mTOR Activation in B[a]P-treated HCoEpC

**Figure 3:**
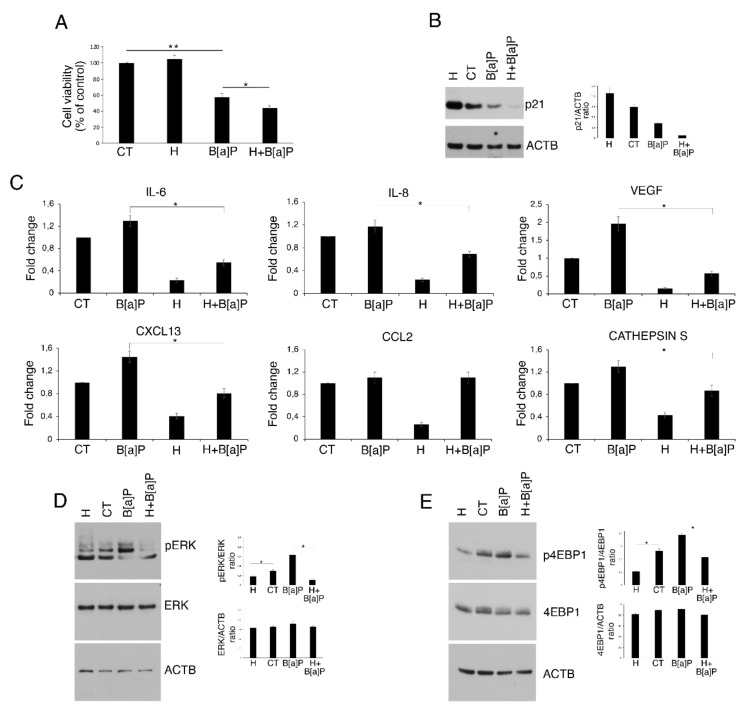
Hydroxytyrosol (H) slightly increases cell death while interferes with the pro-inflammatory milieu formation and ERK and mTOR activation induced by B[a]P treatment of HCoEpC. (**A**) % of cell viability of HCoEpC treated with H (1 μM) and/or B[a]P (5 μM) for 6 days. Mean ± standard deviation (SD) of three independent experiments is shown. (*) *p* < 0.05. (**B**) p21 level in HCoEpC treated with H (1 μM) and/or B[a]P (5 μM) for 6 days was assessed by Western blotting. (**C**) Fold change relative to control of IL-6, IL-8, VEGF, CXCL13, CCL2 and Cathepsin S released by HCoEpC treated with H (1 μM) and/or B[a]P (5 μM) for 6 days. Mean ± standard deviation (SD) of three independent experiments is shown. (*) *p* < 0.05. (**D**) Phospho-ERK 1/2 (pERK) and (**E**) phospho-4EBP1 (p4EBP1) levels in HCoEpC treated with H (1 μM) and/or B[a]P (5 μM) for 6 days were analyzed by Western blotting. Actin (ACTB) was used as loading control and one representative experiment out of three is shown. Histograms represent the mean plus SD of the densitometric analysis of the ratio p21/ACTB, pERK/ERK, ERK/ACTB, p4EBP1/4EBP1 and 4EBP1/ACTB of three different experiments. (*) *p* < 0.05. Untreated cells were used as control (CT) in all the experiments in the figure.

Hydroxytyrosol (H), also known as DPE, is a polyphenol contained in the olive oil that has been shown by ours [30] and other’s laboratories [31] to exert a cytotoxic effect against colon cancer cells. Here, we performed an MTT assay to assess whether this compound could affect the cell viability of HCoEpC cultured in the presence, or not, of B[a]P (5 μM) for 6 days. As shown in Figure 3A, Hydroxytyrosol (1 μM; H) was not cytotoxic for HCoEpC when used alone; rather, cell viability showed a mild increase compared to the untreated control cells (CT). Conversely, we observed that it slight further reduced cell viability (about 10%), in combination with B[a]P (H+ B[a]P) following 6 days of culture, compared to B[a]P-treated cells (B[a]P). Interestingly, Western blot analysis showed a slight decrease in p21 level in cells cultured with H in combination with B[a]P (H+B[a]P), while p21 upregulation was detectable in Hydroxytyrosol-treated (H) compared to untreated (CT) HCoEpC (Figure 3B).

Next, given the antioxidant and anti-inflammatory properties of Hydroxytyrosol, here we evaluated the possibility to counteract the effects induced by B[a]P on HCoEpC by using this compound. We found that Hydroxytyrosol was able to reduce both the constitutive cytokine and chemokine release and the one induced by B[a]P, with the exception of CCL2 (Figure 3C). Moreover, it efficiently counteracted B[a]P-mediated ERK1/2 and 4EBP1 phosphorylation (Figure 3D,E). Altogether, these results indicate that Hydroxytyrosol in combination with B[a]P, by further downregulating p21 level, reduces HCoEpC viability, and, on the other hand, counteracts cytokines and chemokines release as well as the activation of ERK1/2 and mTOR pathways.

### 3.4. Hydroxytyrosol Restores B[a]P-mediated Reduction in Autophagy and Mitophagy in HCoEpC 

**Figure 4:**
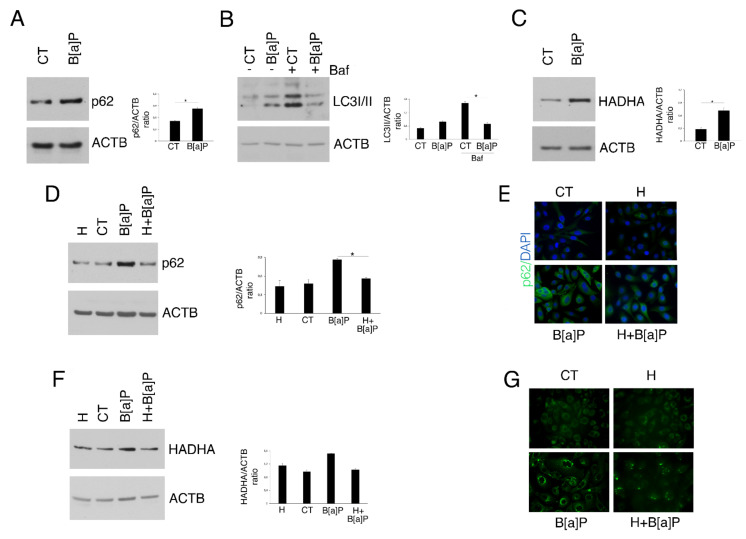
Hydroxytyrosol (H) rescues the B[a]P-mediated impairment of autophagy and mitophagy in HCoEpC. (**A**) SQSTM1/p62 (p62) level in HCoEpC treated with B[a]P (5 µM) for 6 days was assessed by Western blotting. (**B**) LC3II level in HCoEpC treated with B[a]P (5 µM) for 6 days in presence or not of Bafilomycin A1 (Baf; 20 nM for further 4 h) was evaluated by Western blotting. (**C**) HADHA amount in HCoEpC treated with B[a]P (5 µM) for 6 days assessed by Western blotting. (**D**,**E**) SQSTM1/p62 (p62) expression in HCoEpC treated with H (1 µM) and/or B[a]P (5 µM) for 6 days was analyzed by Western blotting and IFA. Nuclei were stained with DAPI. IFA was visualized by fluorescence microscopy, at 20× magnification. (**F**) HADHA in HCoEpC treated with H (1 µM) and/or B[a]P (5 µM) for 6 days evaluated by Western blotting. (**G**) To stain mitochondria, HCoEpC treated with H (1 µM) and/or B[a]P (5 µM) for 6 days were incubated with MitoTracker Green and analyzed by ApoTome system (magnification 40×). In all Western blots showed in the figure, Actin (ACTB) was used as loading control and one representative experiment out of two/three is shown. The histograms represent the mean plus SD of the densitometric analysis of the ratios p62/ACTB, LC3II/ACTB and HADHA/ACTB of three different experiments. (*) *p* < 0.05. Untreated cells were used as control (CT) in all the experiments in the figure.

The reduction in bulk and selective autophagy, particularly mitophagy, has been demonstrated to facilitate cancer onset, mostly due to the accumulation of toxic materials and ROS [32]. Moreover, although with controversial results, these degradative processes have been linked to inflammation [33] and to the activation of pathways such as mTOR [19] and ERK1/2 [20]. Therefore, here we next investigated the autophagic flux by analyzing the expression level of SQSTM1/p62 (p62) and LC3I/II autophagic markers in B[a]P-treated HCoEpC. We found that B[a]P led to p62 accumulation (Figure 4A) and to a reduction in LC3II level (Figure 4B). The latter was evaluated in the presence of Bafilomycin A1, since this drug impairs the lysosomal degradation of LC3II, allowing to assess its formation. These experiments suggest that B[a]P was able to reduce the autophagic flux in these cells. We then also found that mitophagy was impaired by this carcinogen, based on the accumulation of HADHA (Figure 4C), a molecule mainly degraded through mitophagy [34]. Interestingly, the pre-treatment with Hydroxytyrosol was able to restore both pathways. Indeed, both Western blotting analysis and IFA showed a reduction in SQSTM1/p62 (p62) in H+ B[a]P-treated HCoEpC compated to B[a]P alone (Figure 4D,E). In addition, mitophagy was restored by Hydroxytyrosol, as demonstrated by the reduction in HADHA, observed in Western blotting, and by the reduction in mitochondria accumulated following B[a]P treatment (Figure 4F,G).

Thus, these data indicate that Hydroxytyrosol could also efficiently revert these pro-tumorigenic effects induced by B[a]P.

## 4. Discussion

Colon cancer represents a leading cause of death in industrialized countries, due to its high incidence and mortality [35], as in the advanced state it remains an almost incurable cancer. Both genetic and environmental factors represent major risks, and epidemiological studies revealed diet as an important player in this cancer [36,37]. Indeed, red-meat- and fat-rich diets, as well as the presence of carcinogens contained in certain types of food, are associated to an increased colon cancer risk [38]. Benzo[a]pyrene (B[a]P) is a common environmental and food contaminant, whose increased concentration in food is linked to the cooking methods [38]. B[a]P is thought to be involved in colon carcinogenesis [39] and in this study, for the first time, we exposed primary human colonic epithelial cells (HCoEpC) to a B[a]P concentration falling in the assumption range through diet [24]. According to previously reported in vitro [24,25] and in vivo studies [26], we observed a partial cytotoxic effect of this molecule that correlated with a reduction in p21 in HCoEpC, as p21 inhibitor UC2288 lowered HCoEpC cell viability. The reduction in p21 detected in HCoEpC treated by B[a]P could be due to a post-translational regulation; indeed, B[a]P belongs to polycyclic aromatic hydrocarbon carcinogens (PAHs) that have been demonstrated to promote p21-ubiqutination and proteasomal degradation in other cell types [40].

A link between diet and colon cancer development could be also represented by inflammation [41,42], a response physiologically protective and important for tissue homeostasis, which may be pro-tumorigenic when it lasts for a long time and becomes chronic [43]. Inflammatory bowel disease (IBD), including Chron’s disease and ulcerative colitis, represents one of the clearest examples of how inflammation and carcinogenesis are inter-connected [41].

Another process whose dysregulation has been shown to play a role in promoting cancer onset [44], including that of the colon [45], is the reduction in autophagy. This degradative pathway may play a different role, either as a tumor-suppressor or tumor-promoter, depending on the stage of tumor development. It seems to prevent the first steps of carcinogenesis while it promotes cancer progression and survival in response to therapies in established cancers [46,47]. Moreover, a proper regulation of its selective form, called mitophagy, is essential for the elimination of damaged mitochondria that represent the main source of ROS, key molecules in the induction of DNA damage [48].

Here, we found that all of the above-described pro-carcinogenic effects were induced by B[a]P in primary human colonic epithelial cells. Indeed, B[a]P increased the production of pro-inflammatory cytokines, sustained the activation of pro-inflammatory pathways and impaired the autophagic and mitophagic flux. Of note, we revealed that the inflammatory cytokines were able to activate macrophages that, in turn, could amplify inflammation, resulting in a tumor-promoting microenvironment that could exacerbate the risk of cancer development [9,49]. Among pro-inflammatory pathways, we investigated ERK1/2 and mTOR, as the first one has been demonstrated to be involved in several inflammatory disorders, such as psoriatic arthritis (PsA) or rheumatoid arthritis (RA) and IBD [15,50,51], while mTOR was reported to be constitutively activated in active celiac disease (ACD) as well as IBD [13,52].

Interestingly, in this study we found that the pro-tumorigenic effects induced by B[a]P could be efficiently counteracted by pre-treatment with Hydroxytyrosol (also called DPE), suggesting that this natural compound, contained in olive oil, could play an important role in preventing tumorigenesis by chemical carcinogens such as B[a]P. Interestingly, as B[a]P and olive oil are contained in food and can both come into direct contact with colonic epithelial cells, it is important to evaluate their effects, either as single agents or in combination, on normal primary epithelial cells. DPE has been demonstrated by our laboratory to be toxic against colon cancer cells and safe for normal colonic epithelial cells [30]; therefore, its use can be even further encouraged and extended not only to the prevention of colon cancer but also to its treatment.

## 5. Conclusions

Although the ability of olive oil in preventing colon carcinogenesis mediated by B[a]P has been previously demonstrated in vivo [53,54], the present study unveils new molecular mechanisms through which it may occur and shows that one of the most powerful antioxidant compounds contained in olive oil, Hydroxytyrosol, may help to reduce the dysregulation of a number of mechanisms promoting tumorigenesis induced by the carcinogen B[a]P in primary human colonic epithelial cells. These findings encourage further studies aimed at increasing its stability for a more effective clinical application of this natural compound.

## Data Availability

The datasets generated and analyzed during the current study are available from the corresponding author upon reasonable request.
